# Comprehensive Assessment of 16S rRNA Gene Amplicon Sequencing for Microbiome Profiling across Multiple Habitats

**DOI:** 10.1128/spectrum.00563-23

**Published:** 2023-04-27

**Authors:** Wenke Zhang, Xiaoqian Fan, Haobo Shi, Jian Li, Mingqian Zhang, Jin Zhao, Xiaoquan Su

**Affiliations:** a College of Computer Science and Technology, Qingdao University, Qingdao, China; b Shouguang Hospital of Traditional Chinese Medicine, Weifang, China; Chengdu University

**Keywords:** 16S rRNA, amplicon, microbiome, database

## Abstract

The 16S rRNA gene works as a rapid and effective marker for the identification of microorganisms in complex communities; hence, a huge number of microbiomes have been surveyed by 16S amplicon-based sequencing. The resolution of the 16S rRNA gene is always considered only at the genus level; however, it has not been verified on a wide range of microbes yet. To fully explore the ability and potential of the 16S rRNA gene in microbial profiling, here, we propose Qscore, a comprehensive method to evaluate the performance of amplicons by integrating the amplification rate, multitier taxonomic annotation, sequence type, and length. Our *in silico* assessment by a “global view” of 35,889 microbe species across multiple reference databases summarizes the optimal sequencing strategy for 16S short reads. On the other hand, since microbes are unevenly distributed according to their habitats, we also provide the recommended configuration for 16 typical ecosystems based on the Qscores of 157,390 microbiomes in the Microbiome Search Engine (MSE). Detailed data simulation further proves that the 16S amplicons produced with Qscore-suggested parameters exhibit high precision in microbiome profiling, which is close to that of shotgun metagenomes under CAMI metrics. Therefore, by reconsidering the precision of 16S-based microbiome profiling, our work not only enables the high-quality reusability of massive sequence legacy that has already been produced but is also significant for guiding microbiome studies in the future. We have implemented the Qscore as an online service at http://qscore.single-cell.cn to parse the recommended sequencing strategy for specific habitats or expected microbial structures.

**IMPORTANCE** 16S rRNA has long been used as a biomarker to identify distinct microbes from complex communities. However, due to the influence of the amplification region, sequencing type, sequence processing, and reference database, the accuracy of 16S rRNA has not been fully verified on a global range. More importantly, the microbial composition of different habitats varies greatly, and it is necessary to adopt different strategies according to the corresponding target microbes to achieve optimal analytical performance. Here, we developed Qscore, which evaluates the comprehensive performance of 16S amplicons from multiple perspectives, thus providing the best sequencing strategies for common ecological environments by using big data.

## INTRODUCTION

The microbiome plays important roles in various fields such as environment protection (1), bioenergy (2), food (3), and human health (4, 5). In the past decade, amplicon sequencing of the 16S rRNA gene has been regarded as a fast and low-cost approach to studying the microbiome composition and diversity; hence, it has been employed in numerous works such as the Human Microbiome Project (HMP) (6–8), Earth Microbiome Project (EMP) (9), and Metagenomics of the Human Intestinal Tract (MetaHIT) (10). However, people are also concerned about the precision of 16S short reads in profiling due to amplification bias, length limitation of next-generation sequencing (NGS), and insufficient annotation of widely used reference databases (11–13). For example, the 16S rRNA gene has always been considered to only have genus-level resolution (14), for it exhibited a shortage in distinguishing several individual taxa (15). On the other hand, increasing numbers of full-length 16S rRNA gene references have already been produced and recorded in the current databases (16, 17). Since the performance of 16S amplicons in profiling has not been fully tested yet, its resolution on a broad range is still not clear.

Massive data sets suggested that microbiome structures are intensively distinct across habitat types (18), causing microbes that are unevenly distributed (9). For instance, *Firmicutes* and *Bacteroidetes* dominate the human gut microbiota, while *Proteobacteria* and *Cyanobacteria* are prevalent in natural environments (19, 20). Different microbes have their preference in the 16S variable region in terms of amplification sensitivity and nucleotide sequence recognizability. Therefore, based on our previous knowledge of the global beta diversity pattern (19), it is possible to further improve the 16S-based profiling by optimizing configurations in variation region selection, sequencing strategy, and read length.

In recent years, shotgun whole-genome sequencing (WGS) (21, 22) and third-generation full-length 16S rRNA amplicon sequencing technology have enabled species- and strain-level annotation on the microbiome (14, 23), while the requirements of biomass amount, sequencing cost, analysis time, and storage space have also been raised 1 to 2 orders of magnitude higher. More importantly, since a huge amount of the microbiome has been surveyed by 16S amplicon sequencing (over 500,000 samples produced by thousands of studies, stored in open repositories like Qiita [24], MSE [25], and NCBI [26]), some of them were difficult to resample or resequence (e.g., specimens of longitudinal cohorts [27] or those collected from deep-sea sediment [28]). Thus, a comprehensive evaluation of the 16S amplicon short-read sequence in profiling, as well as the continuous update and optimization of this approach, is of significant concern regarding the reusability of previous data, which is also meaningful for guiding further studies on microbiomes.

## RESULTS

### Overall precision of 16S rRNA gene amplicon sequencing in taxonomy profiling.

Briefly, we performed the *in silico* whole procedure of simulation of amplicon production and close-reference taxonomy annotation to measure the performance of microbiome profiling by using the 16S rRNA gene. The amplicon short reads were produced from all the already-known Archaea and Bacteria complete genomes in NCBI RefSeq (29) (239,905 genomes from 35,889 species, which contain 511,460 16S rRNA gene copies in total; refer to Materials and Methods for details). Rather than previous studies that used sliding windows or direct fetch of specific variable regions from reference sequences, here, we simulated the amplification procedure by fragment extraction using primers, which was more like the actual process. Primers covered the V1 to V9 regions (Tables 1 and 2), and lengths of short reads were set as 100 bp, 150 bp, 250 bp, and 300 bp, which have been widely used in NGS for microbiome survey. Sequencing errors were also introduced based on the Illumina data model (refer to Materials and Methods for details). All amplified short reads were then mapped to reference databases in both single-end (SE; only the first end) and paired-end (PE; merged by two ends) types by VSEARCH (30) for taxonomy annotation based on the best hits. We employed three widely used 16S databases, including Greengenes (11) (version 13-8), Silva (13) (version 123), and curated RefSeq 16S rRNA gene database (29) (independent from the RefSeq whole genomes for short-read simulation; refer to Materials and Methods for details) as references (Table 3).

**TABLE 1:** Amplification primers used in this study^*a*^

Primer^*a*^	Sequence^*b*^	Length (bp)	Reference(s)
8F	AGRGTTYGATYNTGGCTCAG	20	42
341F	CCTACGGGNGGCWGCAG	17	43
515F	GTGYCAGCMGCCGCGGTAA	19	44, 45
U789F	TAGATACCCNSGTAGTCC	18	46
967F	CAACGCGAAGAACCTTACC	19	47
1099F	GYAACGAGCGCAACCC	16	48
357R	CTGCTGCCTYCCGTA	15	49
518R	TTACCGCGGCKGCTGGCAC	19	42
806R	GGACTACNVGGGTWTCTAAT	20	44, 45
926R	CCGYCAATTYMTTTRAGTTT	20	42
1064R	CGACRRCCATGCANCACCT	19	50
1406R	GACGGGCRGTGWGTRCA	17	51
1492R	TACCTTGTTACGACTT	16	52

a F, forward primer; R, reverse primer.

b R, A/G; Y, C/T; M, A/C; K, G/T; S, G/C; W, A/T; H, A/T/C; B, G/T/C; V, G/A/C; D, G/A/T; N, A/G/C/T.

**TABLE 2:** Primer sets used for 16S rRNA gene amplification

Amplified 16S rRNA sequence(s)	Forward	Reverse
Full-length	8F	1492R
V1 SE 100 bp, V1 SE 150 bp, V1 SE 300 bp, V1-V2 PE 250 bp	8F	357R
V1-V3 PE 300 bp	8F	518R
V3 PE 150 bp	341F	518R
V3 SE 100 bp, V3 SE 150 bp, V3 SE 300 bp, V3-V4 PE 250 bp	341F	806R
V3-V5 PE 300 bp	341F	926R
V4 PE 150 bp	515F	806R
V4 SE 100 bp, V4 SE 150 bp, V4 SE 300 bp, V4-V5 PE 250 bp	515F	926R
V4-V6 PE 300 bp	515F	1064R
V5 PE 100 bp	U789F	926R
V5 SE 100 bp, V5 SE 150 bp, V5 SE 300 bp, V5-V6 PE 150 bp	U789F	1064R
V6 PE 100 bp	967F	1064R
V6 SE 100 bp, V6 SE 150 bp, V6 SE 300 bp, V6-V8 PE 250 bp	967F	1406R
V7 SE 100 bp, V7 SE 150 bp, V7 SE 300 bp, V7-V8 PE 250 bp	1099F	1406R
V7-V9 PE 250 bp	1099F	1492R

**TABLE 3:** Characteristics of 16S rRNA gene reference databases

Characteristic	RefSeq	Greengenes, 97% cluster	Greengenes, 99% cluster	Silva
No. of sequences	101,484	99,322	203,452	152,265
Clustering threshold (%)	100.00	97.00	99.00	97.00
No. of species annotations	33,175	7,089	4,980	1,230
Proportion of sequences with species-level annotations (%)	100.00	9.37	11.75	31.95

**FIG 1:**
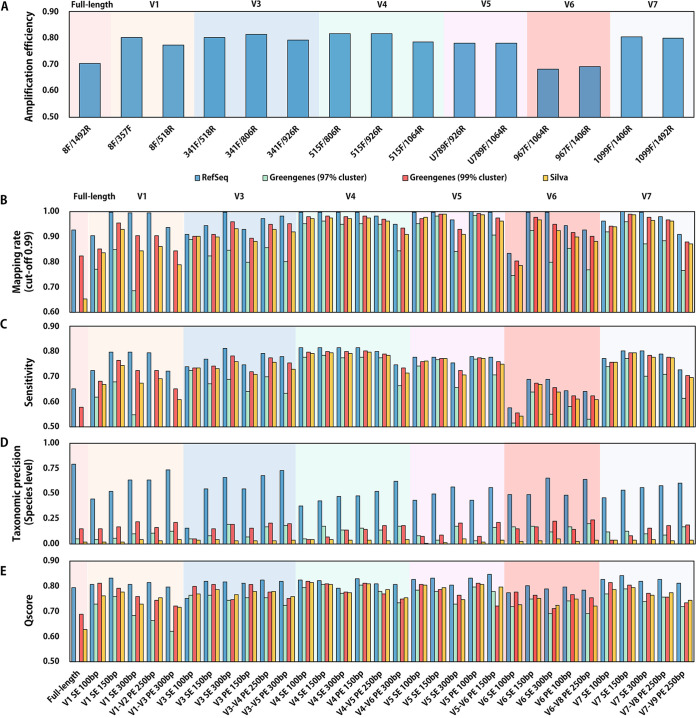
Overall performance of 16S rRNA gene amplicon sequencing with different sequencing strategies. (A) Amplification efficiency of primer sets. (B) Mapping rate to reference databases. (C) Sensitivity of different amplification configurations. (D) Annotation precision at the species level. (E) Qscores of different amplification configurations. Source data are available in Table S1 in the supplemental material.

Figure 1A shows that the 515F/806R primer set had the highest amplification efficiency, 81.72% (417,965 of 511,460). The alignment results in Fig. 1B and D indicate that the RefSeq database also exhibited better recall (mapping rate) and precision (at the species level) than the other two databases. Specifically, using RefSeq as a reference, paired-end 150-bp reads amplified from the V4 region achieved the best sensitivity, 81.57%, to the reference databases (Fig. 1C), and paired-end 300-bp reads amplified from the V1-V3 region had the highest species-level precision, 73.40% (Fig. 1D), which was even close to that of the full-length 16S rRNA gene (78.86%). On the other hand, the low precision of Greengenes and Silva was mainly due to the inconsistent and incomplete annotation in the reference databases. For example, some taxa were not matched with the International Code of Nomenclature of Prokaryotes (ICNP) (31, 32), and a huge number of sequences lacked annotations at the species and even genus levels (33). In addition, we also noticed that paired-end sequencing had advantages in precision for its longer sequence length, while it also suffered from loss of sensitivity from end-merging failure and higher sequencing errors.

Therefore, to comprehensively assess the performance of different amplifying and sequencing strategies, we propose Qscore by considering the amplification efficiency, mapping sensitivity, the precision of multitier taxonomic annotation, sequence type, and length (refer to Materials and Methods for a detailed calculation procedure). The Qscores ranged from 0% to 100%, and a higher value indicated better overall performance. Thus, by integrating the above-described results using Qscore, here, we conclude that paired-ended 150-bp sequencing of the V5-V6 region using the RefSeq database is an optimal method (Qscore = 84.64%) (Fig. 1E) at the “global” scope, which balances amplification and mapping rate, taxonomy precision, and sequencing cost. Also, commonly used strategies of V4 PE 150 bp (83.07%; this also achieves the best average Qscore across all reference databases) and V3-V4 PE 250 bp (82.35%) also obtain high Qscores for profiling (Fig. 1E; see Table S1E in the supplemental material).

### Performance of 16S rRNA gene amplicon sequencing across multiple habitats.

**FIG 2:**
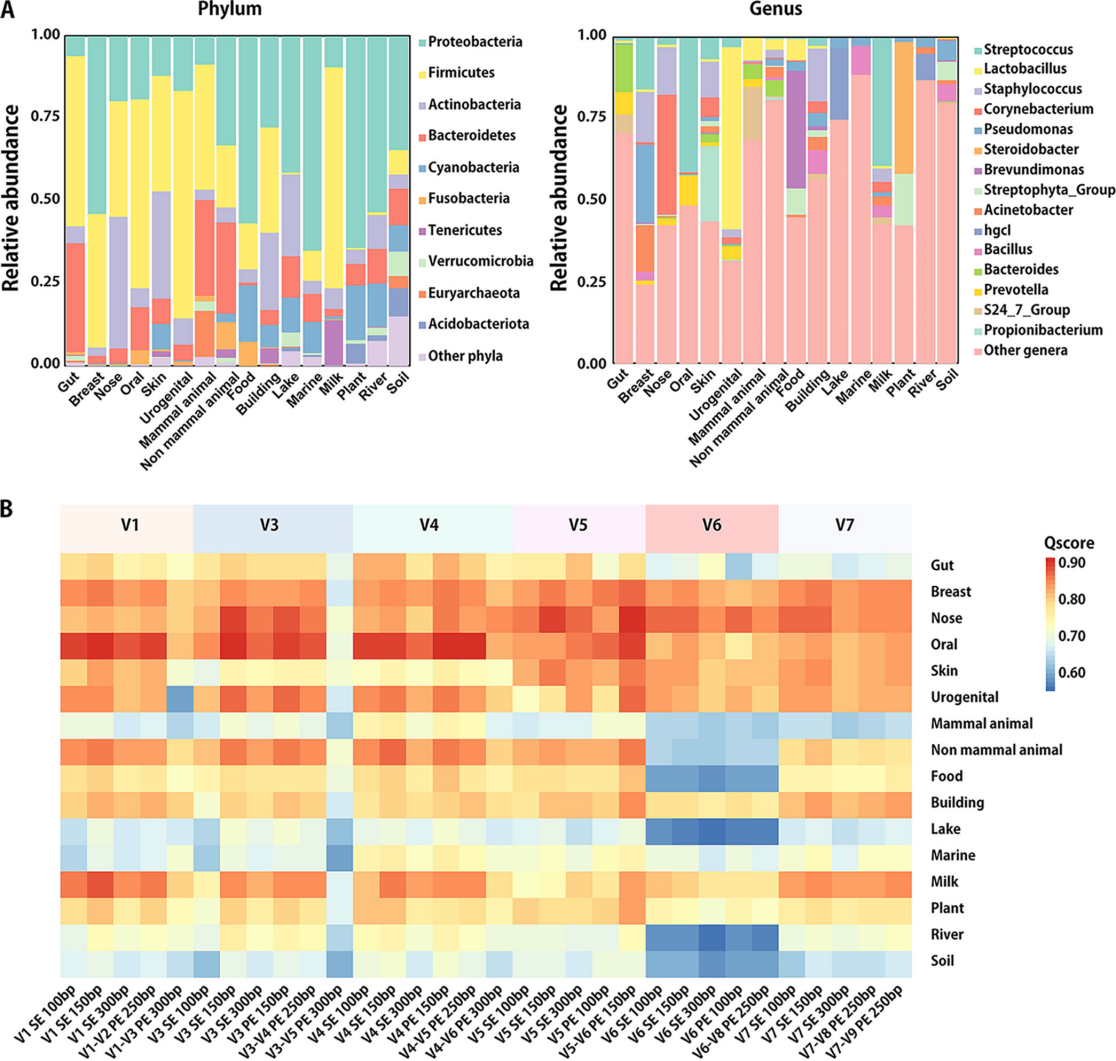
Microbial distribution and Qscores for various habitat types. (A) Taxonomic composition at the phylum and genus levels. (B) Heatmap of Qscores across habitats.

Massive data sets suggested that microbiome structures are intensively distinct across habitat types (9, 18). Different microbes have their preference for the 16S rRNA gene variable region in amplification sensitivity and nucleotide sequence recognizability. To explore the performance of 16S-based profiling among different ecosystems, we downloaded a total number of 157,390 16S amplicon specimens sampled from 16 types of habitats in the MSE (25). By analyzing the distribution patterns of taxonomic compositions (Fig. 2A; Table 4; refer to Materials and Methods for details), we compared their Qscore values (Fig. 2B) and summarized the recommended sequencing strategy for each specific ecosystem.

**TABLE 4:** Number of samples of different habitat types from MSE

Type	Habitat	No. of samples
Human associated	Gut	47,193
	Breast	675
	Nose	157
	Oral	12,539
	Skin	27,069
	Urogenital	3,214
Animal associated	Mammal animal	29,822
	Nonmammal animal	2,038
Environment	Food	97
	Building	1,688
	Lake	4,164
	Marine	6,780
	Milk	1,589
	Plant	2,068
	River	2,251
	Soil	16,046
Total		157,390

Although paired-end V5-V6 150 bp had the top Qscore at the global scope, e.g., it fits for arbitrary microbiomes with unknown structure, the optimal amplification configuration may vary due to different compositional characteristics in specific ecological habitats. With *a priori* taxonomy distribution information from MSE, Table 5 lists the suggested configurations of 16 ecological environments. For instance, dominated by Firmicutes and Bacteroidetes, the optimal amplification region for the gut microbiome is V4, but the V3 region works better for river specimens with abundant Proteobacteria and Acidobacteriota. This list also contains alternative plans that only consider performance without sequencing cost for each habitat, e.g., for soil, V4-V5 PE 250-bp sequences improved the precision on the species level more than V4 SE 150-bp ones, while they also made the cost higher (Table 5; Fig. S2).

**TABLE 5:** Optimal amplification configurations of 16 habitat types^*a*^

Type	Habitat	Optimal amplification configuration	
		Optimal overall	Regardless of cost
Human associated	Gut	V4 SE 150 bp	V3 SE 300 bp
	Breast	V5-V6 PE 150 bp	V5 SE 300 bp
	Nose	V5-V6 PE 150 bp	V5 SE 300 bp
	Oral	V4 PE 150 bp	V4-V5 PE 250 bp
	Skin	V5 SE 150 bp	V5 SE 300 bp
	Urogenital	V5-V6 PE 150 bp	V5 SE 300 bp
Animal associated	Mammal animal	V4 SE 150 bp	V4-V5 PE 250 bp
	Nonmammal animal	V4 SE 150 bp	V3 SE 300 bp
Environment	Food	V5-V6 PE 150 bp	V3 SE 300 bp
	Building	V5-V6 PE 150 bp	V7-V9 PE 250 bp
	Lake	V3 SE 150 bp	V3 SE 300 bp
	Marine	V4 PE 150 bp	V4-V5 PE 250 bp
	Milk	V1 SE 150 bp	V1 SE 300 bp
	Plant	V5-V6 PE 150 bp	V5-V6 PE 150 bp
	River	V3 SE 150 bp	V3 SE 300 bp
	Soil	V4 SE 150 bp	V4-V5 PE 250 bp

a Qscore values are available in Table S2 in the supplemental material, and detailed sensitivity and precision values are available in Table S3.

### *In silico* comparison of microbiome profiling among different sequencing strategies.

To verify the effect of various amplifying and sequencing methods on microbiome profiling assessed by Qscore, here, we performed *in silico* production of 10 human gut microbiomes with 5,060 gut microbe genomes. 16S rRNA amplicon short reads were generated with different configurations. Species-level taxonomy was annotated by the best hit in RefSeq 16S rRNA gene database using VSEARCH with a cutoff value of 0.99, and richness was also corrected by 16S copy number normalization (refer to Materials and Methods for details). Metagenomic shotgun reads were also simulated using identical genomes for reference and then processed by MetaPhlAn2. CAMI-OPAL (34), which includes completeness, purity, L1 norm error, and weighted UniFrac error, was utilized to quantify the performance of different approaches by comparing the taxonomy profiles derived from short reads to the *in silico* experiment design (denoted as ground truth [GT]).

**FIG 3:**
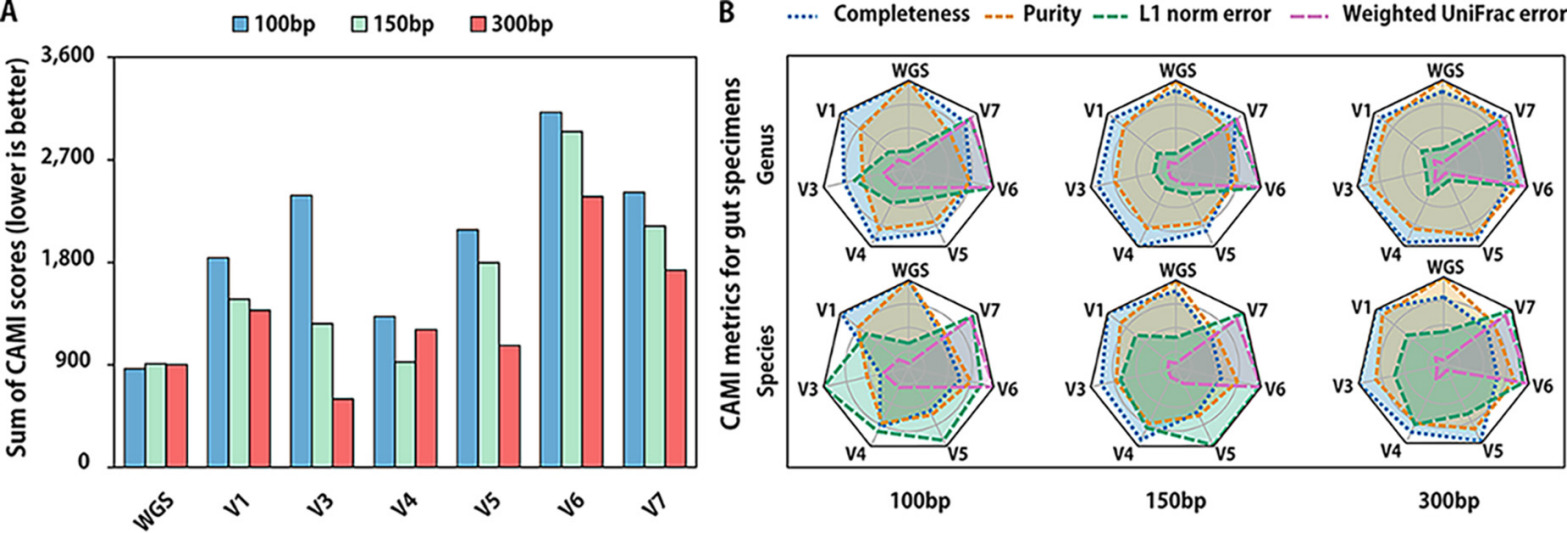
CAMI scores of gut samples simulated by metagenomic sequencing and 16S rRNA sequencing. (A) Sum of CAMI scores. Lower is better. Weights of completeness, purity, L1 norm error, and weighted UniFrac error were set as 1:1:1:1. (B) Detailed scores of completeness, purity, L1 norm error, and weighted UniFrac error of CAMI.

From the CAMI metrics results in Fig. 3, we noticed that the WGS shotgun obtained the best scores for the ground truth. Although there was a significant disparity of CAMI scores among different amplification strategies, V4 SE 150 bp and V3 SE 300 bp achieved the highest performance as the Qscore suggested (Table 5), which produced very close profiles to those by WGS and the ground truth at the species level.

**FIG 4:**
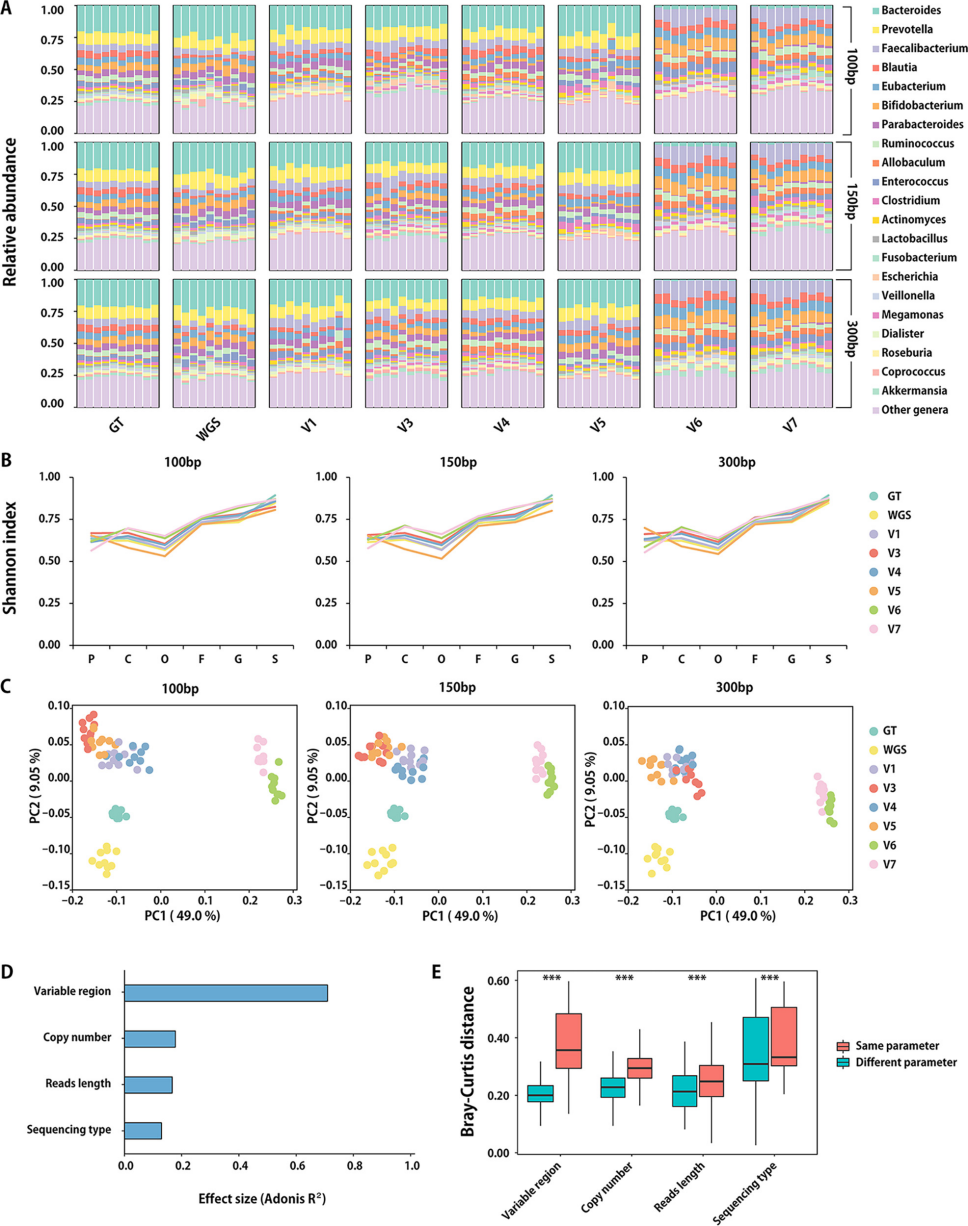
Microbiome profiles and diversity of gut samples simulated by metagenomic sequencing and 16S rRNA sequencing. (A) Taxonomic composition at the genus level. (B) Alpha diversity by Shannon index. (C) Beta diversity of principal-coordinate analysis (PCoA) using Bray-Curtis distances (average of all taxonomic levels). (D) Effect size of sequencing parameters. (E) Bray-Curtis distance between samples with different parameters. ***, *P < *0.01.

The detailed alpha and beta diversity analysis also demonstrated the taxonomy profiles derived from multiple approaches. In Fig. 4A, compositions from 16S V1, V3, V4, and V5 regions were similar to the experiment design and WGS, while V6 and V7 exhibited huge divergence with other configurations. These results were consistent with the Qscores shown in Fig. 2B. Such deviation was not sufficiently reflected by the Shannon index of alpha diversity (Fig. 4B); however, it can be obviously observed in beta diversity patterns (Fig. 4C). Meanwhile, besides the existence of microbes, their estimated relative abundance also plays important roles in profiling. Hence, we further investigated the effect size of multiple parameters on beta diversity patterns (Fig. 4D and E). Results showed that the amplicon variable regions had the greatest impact on the beta diversity pattern (Adonis *R*^2^ = 0.69, *P < *0.01), followed by copy number correction (Adonis *R*^2^ = 0.15, *P < *0.01) (Fig. S3), sequence read length (Adonis *R*^2^ = 0.14, *P < *0.01), and sequencing type (Adonis *R*^2^ = 0.12, *P < *0.01). Therefore, with a suitable sequencing strategy, amplicons can provide reliable microbiome profiling that is close to WGS at the species level at a low cost.

### Qscore online service.

**FIG 5:**
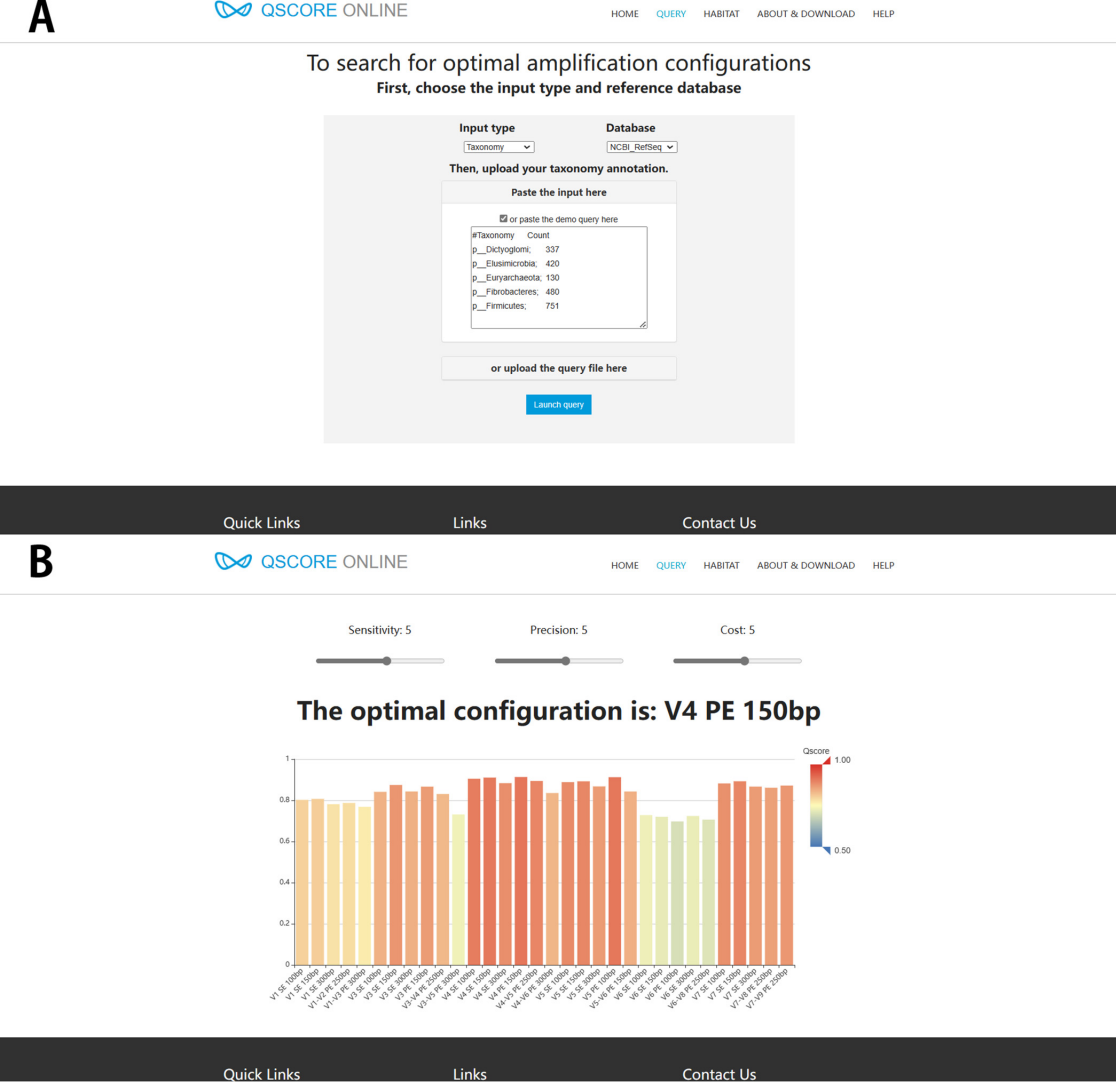
Web service of Qscore. (A) Qscore online accepts two forms of input and provides an example run. (B) Results page of the example run.

To fully exploit the ability and potential of Qscore, we have developed an online service for amplicon sequencing strategy selection at http://qscore.single-cell.cn. With an expected taxonomy pattern (e.g., dominant taxa or microbes of interest), the system can compare different amplification methods and provide the recommended strategy with the highest Qscore. The online Qscore system accepts microbiome compositional features as input, including taxon names (at different levels) or database operational taxonomy unit (OTU; supports reference databases in Table 3). To start an evaluation, users first select the input type from “query by taxonomy” or “query by OTU” (Fig. 5A). The input information can either be uploaded from a plain text file in tabular format or directly pasted into a text box on the webpage. After submitting the query, the results page (Fig. 5B) then returns the Qscore values of different amplification configurations. In default, weights of amplifying and mapping sensitivity, the precision of multilevel taxonomy annotation, and sequencing cost are set as 1:1:1 in Qscore (equation 8), which can also be dynamically customized by users on the website (Fig. 5B).

## DISCUSSION

With the ability to distinguish various microbial species, 16S rRNA amplicon sequencing has worked as a “fingerprint” to study the structure and diversity of microbiomes. By studying the genomes and corresponding 16S fragments of over 35,000 species, we found the amplified short-read sequencing can perform close to the full-length 16S rRNA gene, which achieved 73% precision in species-level classification using sequence alignment against reference databases. Meanwhile, although paired-end sequencing can produce longer reads, it also has more sequencing errors and some sequence read-joining failures, thus reducing the amplification rate and sensitivity.

Since amplicon sequencing can be affected by target gene extraction rate, amplification region, and sequencing length and type, our newly proposed Qscore method fully measures the ability and potential of amplicon short reads in microbial profiling from multiple aspects. We summarized the optimal strategy for 16S amplicon sequencing, which was derived from the analysis of big data, on a universal scope, as well as for 16 commonly studied ecological habitats based on their taxonomic composition patterns. Although there are still deficiencies during the *in silico* procedure, such as the difficulty of repeating the entire random biases of the real experiment, it provides a view of the 16S rRNA gene amplicons from the perspective of big data. In other words, this is also a complement to the real experimental process to guide the design of further studies.

Currently, the 16S-based microbial taxonomy profiling and species recognition are still limited by the shortage of reference databases. For example, the NCBI RefSeq database provides high-resolution annotations at the species level, while Greengenes and Silva have the advantage of comprehensiveness of taxonomy units. On the other hand, functional features inferred from 16S amplicons are helpful to inspire new perspectives for research directions, but the incomplete linkage between amplified target gene and whole-genome functions and heterogeneity among individuals under the same taxonomy unit remain the crucial issue in this stage (35).

## MATERIALS AND METHODS

### Curation and organization of the RefSeq 16S rRNA reference database.

We downloaded 422,034 human-verified 16S rRNA gene sequences of all *Bacteria* and *Archaea* from NCBI RefSeq (https://www.ncbi.nlm.nih.gov/refseq/). Sequences shorter than 1,000 bp were dropped, and then the remainder were deduplicated by CD-HIT (36) clustering with 100% sequence similarity (for duplicated sequences, we only kept the longest one and its annotation). Finally, the RefSeq database kept 101,484 16S rRNA gene sequences from 33,175 species (Table 3; see Fig. S1 in the supplemental material). All sequences were well annotated with full taxonomy from phylum to species. The copy numbers were estimated by the 16S rRNA gene extraction using HMMER (37) (version 3.1; E value < 1e-5) and the pretrained HMM model in Parallel-Meta Suite (38) by SILVA SSU (version 123) (13) from their source genomes to avoid the amplification preference of primers.

### Simulation of 16S rRNA gene amplicons and shotgun metagenomes.

We downloaded the 239,905 complete genomes of 35,889 microbe species from NCBI RefSeq (https://www.ncbi.nlm.nih.gov/refseq/) to simulate short reads of the 16S rRNA gene amplicon and shotgun metagenome. The complete genomes for short-read simulation were independent of the RefSeq 16S rRNA gene reference database.

**(i) Extraction of 16S rRNA gene fragments.** We used the amplicon primer sequences in Table 1 to exactly match the complete genomes for 16S rRNA gene extraction based on regular expressions. First, amplification primers were converted into regular expression strings, e.g., primer 8F, AGRGTTYGATYNTGGCTCAG, was represented by the regular expression of AG[AG]GTT[CT]GAT[CT][ATCG]TGGCTCAG. Such regular expression was then exactly matched among 239,905 whole-genome sequences to identify targeted 16S rRNA gene regions. Specifically, short reads can be generated by extending a fixed length based on the target of primer pairs, e.g., V1V2 PE 250 bp can be obtained by reading 250 bp backward from the match of 8F primer and 250 bp forward from 357R primer simultaneously. Since a genome may contain multiple copies of the 16S rRNA gene, by merging targets of all primers, we obtained a total number of 511,460 16S rRNA gene copies from 239,905 complete genomes.

**(ii) Simulation of 16S rRNA gene amplicons.** With the given primer sets shown in Table 2, we amplified targeted fragments with fixed lengths (100 bp, 150 bp, 250 bp, and 300 bp) and introduced them by sequencing errors in the Illumina data model (39). The quality *Q*(*n*) of the *n*th base in a short read can be generated by equation 1 as follows:

(1) $$Q\left(n\right)=\left(\begin{array}{c} 37-1.88\times 10^{-4}\times n^{2},\;\;5^{\prime }\sim 3^{\prime }\\ 33-1.88\times 10^{-4}\times n^{2},\;\;3^{\prime }\sim 5^{\prime } \end{array}\right)$$

For each base, we generate a random float number in (0, 1). If this number is smaller than $10^{\left[-Q(n)/10\right]}$, this base is randomly replaced by an insertion, deletion, or mutation with probability of 5%, 5%, or 90%, respectively. Here, a total of 30 bases were removed, including a 20-bp primer region at the front side and a 10-bp low-quality region at the end side.

**(iii) Simulation of gut microbiomes.** We simulated 10 human gut microbiomes with 5,060 gut microbe genomes. The genome replication number of each sample is summarized in Table S4. 16S rRNA short reads were generated with different amplification and sequencing configurations. The shotgun metagenomic reads were extracted by a sliding window with a random slide of ~0 to 2,000 bp from the source genomes and cut into fixed lengths (100 bp, 150 bp, and 300 bp). Shotgun metagenomes were only produced by single-end type. Sequencing errors and quality values were introduced in the same way as 16S rRNA gene amplicons. We also removed 20 bp of the low-quality region (10 bp of the front side and 10 bp of the end side) from short reads.

### Taxonomy profiling and annotation.

For amplicon sequences, primers were first removed, and paired-end reads were merged together by FLASH (40). Short reads were annotated by the top hit of sequence alignment to reference databases (Table 3) using VSEARCH (30) (--usearch-global, with cutoff of 0.99) in both single-end (only the first end) and paired-end types. Thus, the results of simulated amplicon reads were named “variable region + sequence type + sequence length.” For example, “V1 SE 150 bp” means 150-bp reads amplified from the V1 region and annotated in the single-end type, and “V1-V3 PE 300” means 300-bp reads amplified from the V1 to V3 regions and annotated in the paired-end type. The relative abundance of species was corrected by the 16S rRNA gene copy number. The taxonomy of metagenomic short reads was annotated by MetaPhlAn 2 (41) with the mpa_v30_CHOCOPhlAn_201901 database using default parameters.

### Calculation of Qscore.

Basically, the Qscore consists of three parts, as follows.

**(i) Sensitivity.** The sensitivity of sequence *i* under the amplification configuration *j* includes both the primer amplification efficiency (PAE) and the sequence matching rate (SMR), which are obtained by equation 2.

(2) completeness(i,j)=PAE(i,j)×SMR(i,j)

Here, both PAE and SMR were also (0, 1) variables depending on if this sequence can be amplified or mapped to the reference.

The average sensitivity of *n* sequences (e.g., a database or a microbial community) under configuration *j* is obtained by equation 3.

(3) $$\text{average sensitivity}\left(j\right)=\overset{n}{\underset{i=1}{\sum }}\text{Abd}\left(i\right)\times \text{completeness}\left(i,j\right)$$

in which Abd(*i*) indicates the relative abundance of sequence *i*.

**(ii) Multitier taxonomy precision.** In taxonomy level *L* (phylum, class, order, family, genus, and species), the precision of multitier taxonomic annotation of sequence *i* under the amplification configuration *j* can be calculated by equation 4.

(4) $$\text{precision}(i,j)=\frac{\sum_{l\in L}{\text{correctness}}_{l}\left(i,j\right)}{\left|L\right|}$$

Here, correctness (*i*, *j*) is a binary variable that denotes whether the taxonomy is correct on level *l* (if the taxonomy does not exist, we set it as incorrect), which is obtained by equation 5.

(5) $${\text{correctness}}_{l}(i,\;j)=\;\left(\begin{array}{c} 0,\;\;\text{if the taxonomy is incorrect on level }l\;\\ 1,\text{ if the taxonomy is correct on level }l \end{array}\right)$$

The average weighted precision of *n* sequences (e.g., a database or a microbial community) under configuration *j* is obtained by equation 6.

(6) $$\text{average precision}(j)\;=\left(\begin{array}{c} \frac{\sum_{i=1}^{n}\text{Abd}\left(i\right)\times \text{precision}(i,j)}{\text{average sensitivity}\left(j\right)},\;\text{ if average sensitivity }\left(j\right)>0\\ \text{0, if average sensitivity}\left(j\right)=0 \end{array}\right)$$

**(iii) Sequencing cost.** For NGS, the sequencing cost is strongly correlated with the read length (Fig. S4; Pearson’s *R*^2^ = 0.9726). Therefore, for amplification configuration *j*, the relationship between amplification cost and sequence length is given by equation 7.

(7) $$\text{cost}(j)=\frac{1,000}{1,000+\text{length}(j)}$$

Hence, for a microbial community with *n* sequences, the performance of 16S amplicon-based profiling under configuration *j* can be assessed by the weighted Qscore as in equation 8.

(8) $$\text{Qscore}(j)=\frac{\left[w_{1}\times \text{average sensitivity}\left(j\right)\right]+\left[w_{2}\times \text{average precision}\left(j\right)\right]+\left[w_{3}\times \text{cost}(j)\right]}{w_{1}+w_{2}+w_{3}}$$

Here, *w_1_*, *w_2_*, and *w_3_* are the weights of the sensitivity, precision, and cost, respectively. In this work, we set them as 1:1:1. In the Qscore website, such weights can also be dynamically customized by users (Fig. 5B).

### Taxonomy profiles of multiple habitats.

We collected 157,390 16S amplicon-based microbiomes of 16 different ecological habitats from the MSE (Table 4). Sequences were clustered into OTUs against the Greengenes database. OTUs with relative abundances of less than 0.01% or sequence count of less than 10 in each specimen were dropped.

For each habitat, we summarized the microbiome composition and distribution by a statistical procedure. Specifically, for an arbitrary OTU *i* of the Greengenes database, its richness can be estimated by equation 9.

(9) $$\text{richness}(i)=\frac{\sum_{j}\text{count}(i,j)}{\sum_{j}\left\{\text{comp}\left[\text{count}(i,j)\right]\times \sum_{i}\text{count}(i,j)\right\}}$$

Here, count(*i*, *j*) is the sequence count of OTU *i* under the amplification configuration *j*, and comp is a function that represents the compatibility of OTU *i* with the primer in configuration *j*, as in equation 10.

(10) $$\text{comp}(x)=\;\left(\begin{array}{c} 0\text{, if}\;x\;\leq \;0\\ 1\text{, if }x>0 \end{array}\right)$$

Finally, we normalized the richness of all OTUs and calculated their relative abundance in equation 11.

(11) $$\text{Abd}(i)=\frac{\text{richness}(i)}{\sum_{i}\text{richness}(i)}$$

### Data availability.

The source code is available at GitHub (https://github.com/qdu-bioinfo/qscore). All data sets are available at the NCBI SRA database under BioProject accession number PRJNA916347.
