# Supplementary material for: Comprehensive Assessment of 16S rRNA Gene Amplicon Sequencing for Microbiome Profiling across Multiple Habitats
Source: Microbiol Spectr. 2023 Apr 27;11(3):e00563-23. doi: 10.1128/spectrum.00563-23 (PMC10269731; doi:10.1128/spectrum.00563-23)
Supplement: Supplemental file 1 — Figures S1 to S4. Download spectrum.00563-23-s0001.pdf, PDF file, 2.7 MB [file spectrum.00563-23-s0001.pdf]

Supplementary Figures

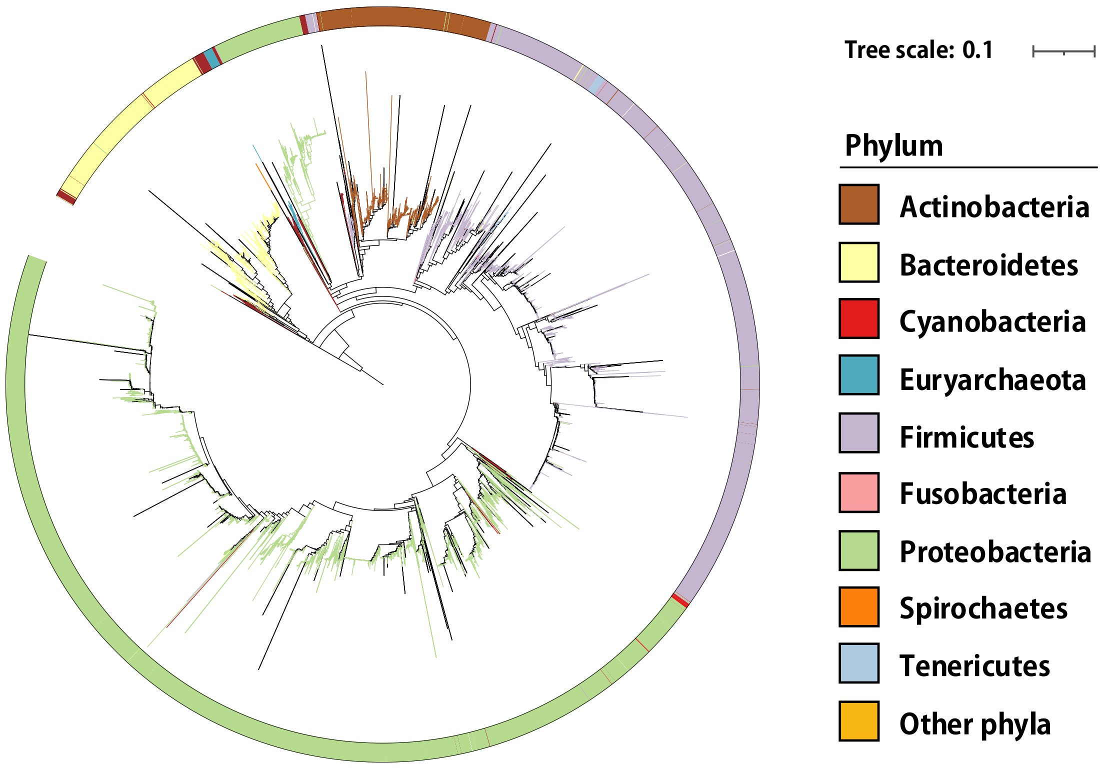

Figure S1. Phylogenetic tree of the RefSeq.

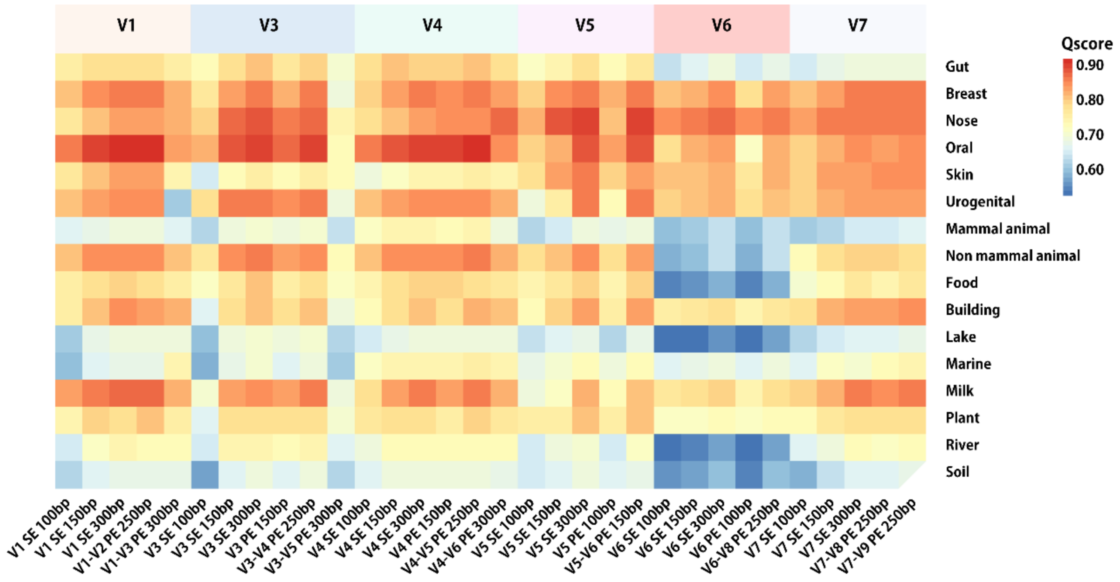

Figure S2. Qscore of regardless of cost for various habitat types. ‘S’ denotes single-ended, and ‘P’ denotes pair-ended.

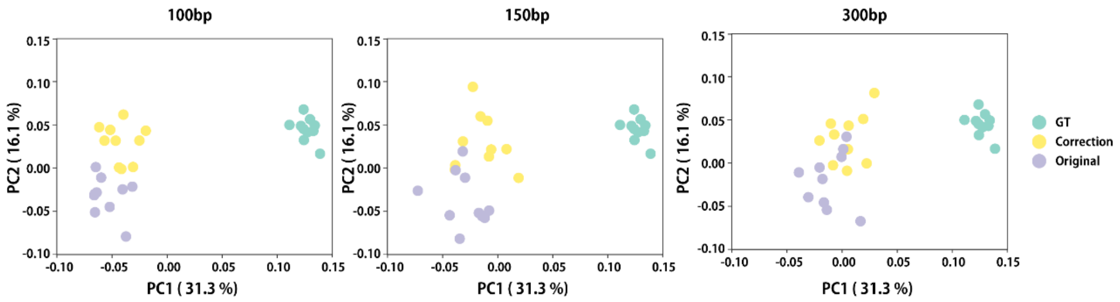

Figure S3. Copy number correction effect.

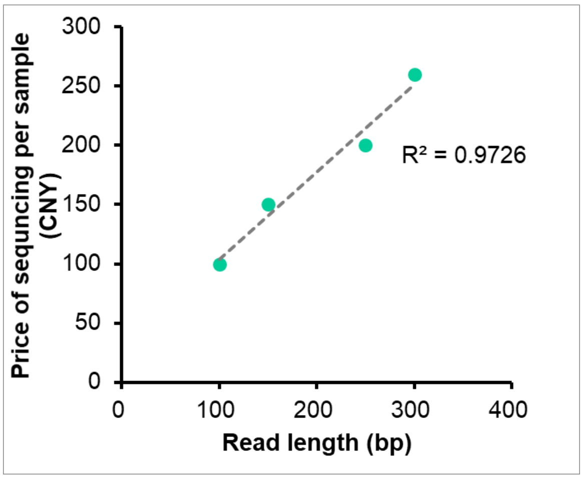

Fig. S4. Correlation between read length and sequencing cost. The sequencing prices were based on surveys from 7 institutes that provide Illumina platforms and solutions.
